# HIV Incidence Estimates Using the Limiting Antigen Avidity EIA Assay at Testing Sites in Kiev City, Ukraine: 2013-2014

**DOI:** 10.1371/journal.pone.0157179

**Published:** 2016-06-08

**Authors:** Ruth Simmons, Ruslan Malyuta, Nelli Chentsova, Iryna Karnets, Gary Murphy, Antonia Medoeva, Yuri Kruglov, Alexander Yurchenko, Andrew Copas, Kholoud Porter, on behalf of CASCADE Collaboration in EuroCoord

**Affiliations:** 1 University College London, London, United Kingdom; 2 Perinatal Prevention of AIDS Initiative, Odessa, Ukraine; 3 Kyiv City AIDS Centre, Ukraine; 4 Public Health England, London, United Kingdom; 5 Institute of Epidemiology, Kyiv, Ukraine; National Institute of Child Health and Human Development, UNITED STATES

## Abstract

**Objective:**

To estimate HIV incidence and highlight the characteristics of persons at greatest risk of HIV in the Ukraine capital, Kiev.

**Method:**

Residual samples from newly-diagnosed persons attending the Kiev City AIDS Centre were tested for evidence of recent HIV infection using an avidity assay. Questions on possible risk factors for HIV acquisition and testing history were introduced. All persons (≥16yrs) presenting for an HIV test April’13–March’14 were included. Rates per 100,000 population were calculated using region-specific denominators.

**Results:**

During the study period 6370 individuals tested for HIV. Of the 467 individuals newly-diagnosed with HIV, 21 had insufficient samples for LAg testing. Of the remaining 446, 39 (8.7%) were classified as recent with an avidity index <1.5ODn, 10 were reclassified as long-standing as their viral load was <1000 copies/mL, resulting in 29 (6.5%) recent HIV infections. The only independent predictor for a recent infection was probable route of exposure, with MSM more likely to present with a recent infection compared with heterosexual contact [Odds Ratio 8.86; 95%CI 2.65–29.60]. We estimated HIV incidence at 21.5 per 100,000 population, corresponding to 466 new infections. Using population estimates for MSM and PWID, incidence was estimated to be between 2289.6 and 6868.7/100,000 MSM, and 350.4 for PWID.

**Conclusion:**

A high proportion of persons newly-infected remain undiagnosed, with MSM disproportionally affected with one in four newly-HIV-diagnosed and one in three recently-HIV-infected. Our findings should be used for targeted public health interventions and health promotion.

## Introduction

Prevalence and incidence rates are important measures of disease. Determining the number of persons who have been recently-infected with HIV enables current transmission patterns to be ascertained, highlighting populations at greatest risk and guiding prevention and intervention strategies. However, given the long latency period which characterises HIV disease, these measures are not easily estimated in practice.

Serological tests identifying biological markers of recent infection are fast becoming an alternative method to cohort studies and mathematical models. These serological tests use key components of the antibody response to the virus, including antibody concentration, response, reaction or proportion, isotype and avidity [1–6]. Supplemented by clinical evidence of a recent or established infection, this process has been termed the Recent Infection Testing Algorithm (RITA). Unlike cohort studies, the RITA methodology requires a sample of serum to be taken at a single point in time; at HIV diagnosis, without the need for follow-up.

Identifying and classifying recent HIV infections in Ukraine is a priority as the country has one of the highest HIV diagnosis rates in Europe at 37.1 per 100,000 population [7]. Furthermore *‘hidden epidemics’* make appropriate targeted public health interventions problematic and, with a shift in HIV risk from persons who inject drugs (PWID) to sexual transmission, particularly heterosexual contact, there is a need to identify and characterise recent HIV infections to better inform policy.

We sought to estimate HIV incidence in the Ukraine capital, Kiev, and to highlight the characteristics of persons at greatest risk of HIV using published methodologies [8,9]. Findings from this study on testing and positivity rates have previously been published [10]. Here we examine factors related to recent infection and provide HIV incidence estimates using data from the same cohort.

## Methods

In April 2013 we introduced a new methodology for data collection for persons newly-presenting for an HIV test at the main testing facilities in the City of Kiev. These testing facilities consisted of four infectious disease clinics for HIV, collectively known as the Kiev City AIDS Centre. Details of this methodology have been described previously [10]. In Brief, at the time of testing we collected information on residence, risk factors for acquiring HIV, reason for test and testing history through a short anonymous questionnaire to be completed by the clinic attendee using a handheld electronic tablet.

All adults (≥16years) presenting or referred for an HIV test at the Kiev City AIDS Centre, between 1st April 2013 and 31st March 2014 were included. Persons testing as part of antenatal or blood donation screening were excluded, as different methodologies for estimating HIV incidence are required for these populations, given that population sizes for these groups are known.

### Data Management

Data were captured by a remote secure server and downloaded for analysis. Records were de-duplicated using year of birth, sex, and identification number, with any discrepancies queried and resolved with the laboratory. The identification number was used to match the person’s data with their HIV test result. All records with incomplete information (year of birth, sex and date of diagnosis), or with a previous HIV positive test and, therefore, not a new diagnosis, were removed. Data are presented for persons aged 16 years and over by age quartiles.

Probable route of exposure was determined based on the attendee’s answer to the following questions: ever injected drugs, ever had sex with a person of the opposite sex, ever had sex with a person of the same sex, ever paid for sex, and ever been paid for sex. Persons with more than one reported risk factor were classified by the following hierarchical order: PWID, men who have sex with men (MSM), then sex between men and women. Additional information was available on sexual behaviour (ever paid for sex, ever been paid for sex, or partner of a PWID).

Information on the reason for test was grouped into the following three categories: clinical indication (persons presenting with symptoms), high risk population (those regarded to engage in high risk behaviour i.e. injecting drugs, contact of a known HIV-positive person, those diagnosed with a sexually transmitted infection, those who experienced an occupational HIV risk, and those reporting sex with multiple partners), and general screening (before surgical interventions, recruitment into the army, prisoners and persons requiring an HIV test due to regulations or policy, e.g. for employment or visa procurement). Persons with more than one reported reason for test were classified by the following hierarchical order: general screening, clinical indication, then high risk population.

### Laboratory Methods

All samples were tested for HIV at the HIV reference laboratory at the Kiev City AIDS Centre, Ukraine using an ELISA test. Residual samples from persons with a confirmed new diagnosis were tested for evidence of recent infection using the limiting antigen (LAg) avidity EIA assay [11–13].

The LAg avidity assay differentiates recent from long-standing infections using the strength of the bond between the viral protein (antigen) and the HIV-specific antibody. A low avidity represents a recent infection. The normalised optical density is estimated by dividing the optical density of the specimen by the mean optical density of the calibrator, with recent infection being assigned to samples with an optical density of <1.5, and a mean duration of recent infection being equal to 130 days (95% CI: 118–142) [13]. The initial test requires samples to be screened, with samples with an ODn ≤2.0 being tested in triplicate for confirmation.

As evaluations of the LAg avidity assay indicated that the assay performed poorly on specimens with a low or undetectable viral load and from persons on cART [14], samples with viral load <1000 copies/mL were reclassified as longstanding.

### Incidence Estimates

HIV incidence was estimated using the stratified extrapolation method initially proposed by Karon *et al* (18) and modified by Prejean *et al* [8]. Briefly, this modified method uses the observed number of recent infections, and the probability a person will present for an HIV test and be classified as recent using RITA, to estimate the true number of infections within the population during the period of interest. The observed number of recent infections equates to the number of HIV-positive individuals testing for HIV and being assigned recent by the LAg assay, after reclassifying samples with viral load <1000 copies/mL as longstanding. To establish the true number of recent infections (Tr) the observed number (Or) is divided by the probability of testing and being classified as recent (P).

Tr=Or/P

The estimation of this probability is derived for repeat testers and first-time testers separately. For repeat testers, i.e. with previous HIV negative test result, P_i_ is estimated as the probability of being classified as recent (Sw(t)) at the time of their test since infection, over the distribution of possible infection time, which is assumed uniform over the interval between the last negative and first positive test dates. For each individual we consider the time in months between their last negative test and first positive test dates. The probability of testing and being classified as recent for that individual (P_i_) is the weighted sum of each possible date of seroconversion within that interval. The overall probability of testing and being classified as recent (P) is then the average of each person’s P_i_.

$$P_{i}=\;\frac{1}{T_{i}}\int_{0}^{Ti}Sw\left(t\right)dt$$

The assumption for first-time testers is that the testing rate is constant during the interval from infection to AIDS (defined as a CD4 cell count <200 cells/mm^3^), where the rate, the estimated mean time from infection to first test (*β*) can be made based on the proportion of persons diagnosed with a simultaneous HIV and AIDS diagnosis referred to as q; *α*_*A*_ being the shape parameter of the incubation period from infection to an AIDS diagnosis, and *β*_*A*_ being the scale parameter of the incubation period.

$$\beta =\frac{\left[\beta_{A}\right]}{\left[q^{-1/\alpha_{A}}-1\right]}$$

P is estimated by the weighted sum of the probability that a person tests within the assay’s recency period, and the probability that the test is performed before AIDS develops.

$$\int_{0}^{\infty }Sw\left(t\right)S_{A}\left(t\right)\frac{1}{\beta }e^{-t/\beta }dt$$

Where data were missing on probable route of exposure, RITA classification or testing history, we used a 20-fold multiple imputation procedure on the observed data and 95% confidence intervals were calculated using bootstrap methodologies, where the original data were resampled 1000 times to create 1000 datasets from which the probabilities for testing and being classified as recent were estimated to give the upper and lower estimates.

Region-specific denominators for Kiev City were based on data collected through the government census [15]. Rates were calculated by dividing the number of tests, diagnoses or estimated number of recent infections for the calendar year by the population denominator and multiplied by 100,000. Incidence rates per year were calculated for Kiev City overall, and by age and sex using population estimates as denominators. We used published estimates of subpopulations at risk of HIV to derive subgroup estimates. The first is from the AIDS Alliance which estimated that, in Kiev City in 2012, there were 23,400 PWID and 9,400 MSM, the latter being 0.9% of the male population [16]. The second is from the European MSM internet survey (EMIS) which estimates that MSM represented 0.3% of the male population, with the ‘best’ estimate being 0.7% [17].

For comparison with published literature we also present incidence estimates by the original method by Karon *et al* [9], where the probability of testing and being classified as recent (P), is the product of the probability of testing within a year of infection (P_1_), the probability of a confirmed positive sample having a RITA result (P_2_) and the probability the result is recent within a year of infection (P_w_). P is estimated as follows.

$$P=\;{\text{P }}_{1}\times \;P_{2}\times P_{W}$$

Statistical analyses were performed using STATA version 12 (STATA Corp, College Station, TX, USA).

### Ethics statement

The study was part of CASCADE within EuroCoord (www.EuroCoord.net) funded by the European Union Framework Programme VII. Ethics approval was given by the ethics committee of the Institute of Epidemiology and Infectious Diseases, Academy of Medical Sciences of Ukraine, Kiev, Ukraine and the ethics committee of University College London (UCL).

## Results

### Population characteristics

During the 12-month period April 2013 –March 2014, 6402 persons were tested for HIV. Thirty-two were excluded as follows: 7 records were missing year of birth, sex or identification number, and 25 persons were already known to be HIV positive.

The remaining 6370 tests (3425 in men and 2945 in women) gave a crude test rate of 293.2 per 100,000 population (aged ≥16 years). HIV test results could not be linked for 353 specimens. Of the remaining 6017 tests with a result, 467 (7.8%) were HIV positive, equivalent to a diagnosis rate of 21.5 per 100,000. The completion rate for questionnaires was 99.4% with 39 persons not providing any further information. The median age among those newly-diagnosed was 32 years [Inter Quartile Range: 28–36], and HIV prevalence among those testing was highest among persons aged 31–35 years, males, PWID and MSM (Table 1). Over half (56%) of persons newly-diagnosed tested due to clinical indicators, 35% due to high risk behaviour, and 8.7% through general public screening. Of note, the majority of persons tested through screening reported more than one reason for test, of whom 33 (83%) also reported high risk behaviour.

**Table 1:** Characteristics of persons newly diagnosed with HIV and the proportion identified as recently infected using the LAg Avidity EIA, Kiev City, Ukraine: April 2013 –March 2014.

	Newly Diagnosed		Samples available for LAg test	Recent Classification	
	N = 467	%	N = 446	N = 29	%
**Age (years)**					
16–25	64	4.6	61	8	13
26–30	120	7.1	115	10	8.7
31–35	154	11.2	144	8	5.6
≥36	129	8.3	126	3	2.4
**Sex**					
Male	303	9.4	288	23	8.0
Female	164	5.9	158	6	3.8
**Residence**					
Kiev (city)	391	7.2	373	28	7.5
Kiev area (small town)	53	13.8	51	1	2.0
Kiev area (village)	10	9.3	10	0	0
Another area of Ukraine	7	16.3	6	0	0
Not Reported	6		6		
**Probable route of exposure**					
Persons who inject drugs: Male	137	17.1	130	3	2.3
Persons who inject drugs: Female	41	21.5	40	1	2.5
Men who have sex with men	46	24.1	43	14	33
Heterosexual contact: Male	114	5.1	109	5	4.6
Heterosexual contact: Female	122	4.7	117	5	4.3
Not Reported	7		7	1	
**Testing History**					
Repeat tester	84	6.2	76	5	6.6
First-time tester	349	7.8	338	22	6.5
Not reported	34		32	2	
**Reason for test**					
Clinical Indicators	257	11.0	248	20	8.1
High risk Behaviour	163	5.5	154	8	5.2
General Public Screening	40	6.0	37	1	2.7
Not Reported	7		7		

### Recent HIV infection

Of the 467 individuals newly-diagnosed with HIV, 21 had insufficient samples for LAg testing. Of the remaining 446, 39 (8.7%) were classified as recent with an avidity index <1.5 ODn. Of those, 10 had a viral load <1000 copies/mL and were, therefore, not considered recent. Therefore, of 446 individuals tested, 29 (6.5%) were recent HIV infections; 6.6% among repeat testers and 6.5% among first-time testers.

For the 29 recently-infected individuals, median age was 30 years [IQR: 24–34], with men accounting for two thirds of recent infections. Table 1 shows the distribution of persons classified as recently-infected, with a higher proportion of recent infections among younger adults, men, MSM, and those resident in Kiev City.

We found that the only independent predictor for being recently-infected was probable route of exposure, with MSM more likely to present with a recent infection compared with persons reporting heterosexual contact [Odds Ratio 8.86; 95% CI 2.65–29.60] (Table 2). Where exposure category was reported, MSM accounted for 50% (14/28) of recent infections with heterosexual men and women each accounting for 18% (n = 10). The majority of MSM classified as recent fell into the age group 26–30 years (57%; 8/14) and tested due to high risk behaviour (71%; 10/14). PWID represented 14% (n = 4) of recent infections, of whom the majority (75%) were male.

**Table 2:** Factors association with testing recent according to the LAg assay in Kiev City, Ukraine: April 2013 –March 2014

	Univariate odds ratio for testing recent (95% confidence interval) pvalue		Multivariate odds ratio for testing recent^¥^ (95% confidence interval) pvalue	
**Age Years (per 10 year increase)**	0.48 (0.25,0.94)	0.023	0.65 (0.32,1.33)	0.217
**Sex**				
Male	1	0.133	1	0.928
Female	0.51 (0.20,1.29)		0.96 (0.28,3.17)	
**Residence**				
Kiev City	1	0.039	1	0.141
Outside Kiev City	0.19 (0.03,1.45)		0.27 (0.03,2.14)	
**Probable route of exposure**				
Persons who inject drugs	0.61 (0.18,2.01)		0.59 (0.16,2.10)	
Men who have sex with men	11.05 (4.39,27.82)		8.86 (2.65,29.60)	
Heterosexual contact	1	<0.001	1	<0.001
**Testing History**				
Repeat tester	1	0.960	1	0.256
First-time tester	1.03 (0.38,2.80)		0.76 (0.25,2.30)	
**Reason for test**				
Clinical Indicators	1	0.236	1	0.621
High risk behaviour	0.57 (0.23,1.38)		0.47 (0.18,1.25)	
General public screening	0.31 (0.04,2.39)		0.46 (0.06,3.80)	

^¥^ Adjusting for all factors in table.

### HIV incidence estimates

We estimated HIV incidence at 21.5 per 100,000 population during the period April 2013 –March 2014, corresponding to 466 new infections.

Although a higher proportion of recent infections were among 16–25 year olds, incidence estimates were highest among those aged 26–30 years and 31–35 years (both 72.5 per 100,000). Men had the highest incidence estimates at 34.3 compared with 10.2 for women. Using population estimates for MSM and PWID, incidence was estimated to be between 2289.6 and 6868.7 per 100,000 MSM, and 350.4 for PWID (Table 3).

**Table 3:** Incidence estimates for Kiev City, Ukraine, April 2013 –March 2014, by subpopulations.

	Population (16)	Number newly infected with HIV (95% CI) ^††^		HIV incidence rate (95% CI) ^†^	
**Total**	2,172,448	466	396–567	21.5	18.2–26.1
**Age (years)**					
16–25	484,839	121	102–148	25.0	21.0–30.5
26–30	212,340	154	130–188	72.5	61.2–88.5
31–35	177,968	129	109–159	72.5	61.2–89.3
≥36	1,297,301	59	50–71	4.5	3.9–5.5
**Sex**					
Male	990,412	340	288–414	34.3	29.1–41.8
Female	1,182,301	120	102–147	10.2	8.6–12.4
**Probable route of exposure**					
Persons who inject drugs	23,400^¥^	82	70–1000	350.4	299.1–427.4
Men who have sex with men	2970^‡^	204	172–251	6868.7	5791.2–8451.2
	8910^‡‡^			2289.6	1930.4–2817.1
Heterosexual contact	≠	159	135–195	≠	≠

^†^ per 100,000 population

^††^ Adjusted for Viral load

^≠^ Population level data not available

^¥^ Based on MARP estimates by AIDS Alliance [18]

^‡^ Derived from the European MSM internet survey (EMIS), where an estimated 0.3% of the male population are MSM [17]

^‡‡^ Based on MARP estimates by AIDS Alliance, where an estimated 0.9% of the male population are MSM [18].

Source: State Statistics Service of Ukraine. Ukraine Census [15].

Incidence estimates using the Karon *et al* methodology results in an incidence estimate of 25.9 per 100,000 with 562 new infections.

## Discussion

For the first time we present direct estimates of HIV incidence for Kiev City for the period April 2013 –March 2014, with 21.5 per 100,000 population, equating to an estimated 466 new HIV infections during that period. These findings are similar to those reported for the USA using the same methodology, where the overall incidence estimate was 19.0.

It is difficult to place our estimate within the global context, due to differences in methodologies used. The extrapolation process for identifying the true number of recent infections in a population has been used in the USA, France, and the Italian region of Lazio, with HIV incidence estimates of 19.0, 17.0, and 19.9, respectively [8,19,20]}. However, estimates for France and Lazio were based on the original methodology by Karon et al. Limitations to the original methodology discussed by Prejean et al [8] suggest that the incidence estimates of 17.0 and 19.9 per 100,000 for France and Lazio, respectively, are an overestimation. This has been demonstrated by the USA where both methodologies were used with incidence decreasing from 22.8 using the original methodology to 19.0 per 100,000 [8,21]. Our incidence estimates using the Karon methodology were 25.9 per 100,000 compared with 21.5. Both the distribution of new diagnoses and recent infections demonstrated disproportionate HIV infection among MSM [10], with one in four MSM newly-diagnosed and one in three recently-infected. The estimated number of new infections among MSM was 185, representing the majority of the 206 estimated for the overall population. Using published estimates [16,17] to define the population at risk within Ukraine, incidence estimates for MSM ranged between 2076.3 new infections per 100,000 MSM based on 0.9% of the male population being MSM [16], and 6229.0 if this proportion was 0.3% [17]. These alarmingly high incidence rates, compared with those for the overall population, are similar to rates given for France [20] (1006 among MSM vs. 17 among the total population) and Los Angeles county [22] (493 vs. 23 respectively). However, these estimates are based on the original methodology by Karon *et al*, which Prejean *et al* highlighted results in an overestimation of incidence.

The work builds on our previous findings indicating that MSM and PWID are disproportionately affected by HIV; we reported that diagnosis rates for these two groups were as high as 24.1% and 17.9%, respectively compared with 4.9% among persons reporting heterosexual contact. Our data also indicated a bridging between high risk individuals and their heterosexual partners with an estimated 1 in 6 heterosexual women reporting contact with a PWID [10].

For the first time detailed information on HIV risk behaviour was available, allowing for targeted awareness, prevention and testing among at-risk populations. This disproportionate distribution of HIV among MSM indicates that onward transmission in this group is high, and highlights the need for tailored prevention and intervention strategies [10]. Furthermore, the lack of exposure information across Ukraine means that the scale of the epidemic among these high risk groups is unknown. Work in the UK on the likely sources of onward transmission in this subpopulation suggests that high incidence among MSM is driven by condomless sex and would be even higher without the introduction of antiretroviral therapy [23]. ART coverage in Ukraine is one of the lowest internationally (22–28%) [24] and EMIS indicated that MSM in Ukraine are not being reached by prevention efforts, and have minimal understanding of HIV and its prevention [17].

Like MSM, PWID are also a hard to reach population, with underlying tension between law enforcements and those involved in public health [25]. Diagnosis figures for Ukraine [7] show sexual contact between men and women exceed that of injecting drug use becoming the leading route of infection. However, there is evidence of onward transmission within this group suggesting that PWID are still an important subpopulation. UNAIDS indicates that harm reduction programmes are available in all regions of Ukraine, but reach only a third of those at risk, and <10% are accessing treatment [26]. Work modelling the effectiveness of medication-assisted treatment for opioid dependence (MAT), ART and syringe exchange programmes (SEP) among PWID, estimated that as high as 40% of HIV among PWID could have been avoided [27].

There are several limitations worthy of discussion. Firstly, the data required to estimate the probability of testing and being classified as recent are prone to error. For repeat testers this is based on the time between the last negative test and an individual’s first positive test which is, therefore, reliant on these data being reported accurately. In Kiev City, as is likely in most surveillance systems, the availability of these data is based on self-reporting. Underestimating the time interval between an individual’s negative and positive test will increase the probability of testing and being classified as recent. A higher probability would result in an underestimation of the true number of recent infections. If this interval is overestimated this would result in a lower probability and an overestimated incidence rate. For first-time testers, the proportion of the population diagnosed late is used to estimate the time between infection and presenting for a test. As availability of these data are limited for Kiev City, we used data collected from patient notes for the first quarter of 2013 and estimated the proportion among all new diagnoses rather than for first-time testers specifically. It is difficult to know how representative the assumptions used within the model among those seeking testing are relative to those who do not. However, the model assumes that all HIV infections will be diagnosed eventually. Secondly, population data, particularly for MSM, within Kiev City was estimated using published literature, based on the work of AIDS Alliance [16] and the European MSM internet survey [17]. There are uncertainties within these estimates, and we present the results based on the minimum and maximum estimates. Further work is needed to establish the likely numbers at risk. Thirdly, data from Kiev City may well not be generalizable to the rest of Ukraine. For a better understanding of the epidemic in other regions, it would be beneficial to consider implementation of our data collection methods across Ukraine. Fourthly, the proportion of persons diagnosed late was not available by subpopulations and, therefore, incidence will have been under or over-estimated for subgroups as the probability of being diagnosed recent for the overall population was applied to subgroup estimates. Finally, we were unable to assess the possible effect of previous ART use on our estimates. It is unlikely, however, that any newly-diagnosed individuals had been on ART given the low ART coverage rate among those in need of it in the Ukraine. In any case, we reclassified all those with a low VL as non-recent, which will have reduced any possible effect of ART.

This study speaks to the need for targeted testing. With only 29 persons diagnosed among the estimated 466 new infections, a high number of persons newly-infected remain undiagnosed, who may well be participating in high-risk behaviour increasing the risk of onward transmission. The transmission rate among undiagnosed persons has been estimated to be 3.5 times higher than among those diagnosed [28], as those undiagnosed are likely to have a higher viral load and, therefore, at higher risk of onward transmission [29–34]. Furthermore, this knowledge base will better enable targeted public health action, health promotion work, laying a foundation to facilitate local and national guidelines to be developed, and to support work being conducted by government and non-government organisations.

## Appendix

**CASCADE Steering Committee**: Julia Del Amo (Chair), Laurence Meyer (Vice Chair), Heiner C. Bucher, Geneviève Chêne, Osamah Hamouda, Deenan Pillay, Maria Prins, Magda Rosinska, Caroline Sabin, Giota Touloumi.

**CASCADE Co-ordinating Centre**: Kholoud Porter (Project Leader), Ashley Olson, Andrea Cartier, Lorraine Fradette, Sarah Walker, Abdel Babiker.

**CASCADE Clinical Advisory Board**: Heiner C. Bucher, Andrea De Luca, Martin Fisher, Roberto Muga

**CASCADE Collaborators**: Australia PHAEDRA cohort (Tony Kelleher, David Cooper, Pat Grey, Robert Finlayson, Mark Bloch) Sydney AIDS Prospective Study and Sydney Primary HIV Infection cohort (Tony Kelleher, Tim Ramacciotti, Linda Gelgor, David Cooper, Don Smith); Austria Austrian HIV Cohort Study (Robert Zangerle); Canada South Alberta clinic (John Gill); Estonia Tartu Ülikool (Irja Lutsar); France ANRS CO3 Aquitaine cohort (Geneviève Chêne, Francois Dabis, Rodolphe Thiebaut), ANRS CO4 French Hospital Database (Dominique Costagliola, Marguerite Guiguet), Lyon Primary Infection cohort (Philippe Vanhems), French ANRS CO6 PRIMO cohort (Marie-Laure Chaix, Jade Ghosn), ANRS CO2 SEROCO cohort (Laurence Meyer, Faroudy Boufassa); Germany German HIV-1 seroconverter cohort (Osamah Hamouda, Claudia Kücherer, Barbara Bartmeyer); Greece AMACS (Anastasia Antoniadou, Georgios Chrysos, Georgios L. Daikos); Greek Haemophilia cohort (Giota Touloumi, Nikos Pantazis, Olga Katsarou); Italy Italian Seroconversion Study (Giovanni Rezza, Maria Dorrucci), ICONA cohort (Antonella d’Arminio Monforte, Andrea De Luca.) Netherlands Amsterdam Cohort Studies among homosexual men and drug users (Maria Prins, Ronald Geskus, Jannie van der Helm, Hanneke Schuitemaker); Norway Oslo and Ulleval Hospital cohorts (Mette Sannes, Oddbjorn Brubakk, Anne-Marte Bakken Kran); Poland National Institute of Hygiene (Magdalena Rosinska); Spain Badalona IDU hospital cohort (Roberto Muga, Jordi Tor), Barcelona IDU Cohort (Patricia Garcia de Olalla, Joan Cayla), CoRIS-scv (Julia del Amo, Santiago Moreno, Susana Monge); Madrid cohort (Julia Del Amo, Jorge del Romero), Valencia IDU cohort (Santiago Pérez-Hoyos); Sweden Swedish InfCare HIV Cohort, Sweden (Anders Sönnerborg); Switzerland Swiss HIV Cohort Study (Heiner C. Bucher, Huldrych Günthard, Martin Rickenbach); Ukraine Perinatal Prevention of AIDS Initiative (Ruslan Malyuta); United Kingdom Public Health England (Gary Murphy), UK Register of HIV Seroconverters (Kholoud Porter, Anne Johnson, Andrew Phillips, Abdel Babiker), University College London (Deenan Pillay); African cohorts: Genital Shedding Study (US: Charles Morrison; Family Health International, Robert Salata, Case Western Reserve University, Uganda: Roy Mugerwa, Makerere University, Zimbabwe: Tsungai Chipato, University of Zimbabwe); International AIDS Vaccine Initiative (IAVI) Early Infections Cohort (Kenya, Rwanda, South Africa, Uganda, Zambia: Pauli N. Amornkul, IAVI, USA; Jill Gilmour, IAVI, UK; Anatoli Kamali, Uganda Virus Research Institute/Medical Research Council Uganda; Etienne Karita, Projet San Francisco, Rwanda).

**EuroCoord Executive Board**: Fiona Burns, University College London, UK; Geneviève Chêne, University of Bordeaux, France; Dominique Costagliola (Scientific Coordinator), Institut National de la Santé et de la Recherche Médicale, France; Carlo Giaquinto, Fondazione PENTA, Italy; Jesper Grarup, Region Hovedstaden, Denmark; Ole Kirk, Region Hovedstaden, Denmark; Laurence Meyer, Institut National de la Santé et de la Recherche Médicale, France; Heather Bailey, University College London, UK; Alain Volny Anne, European AIDS Treatment Group, France; Alex Panteleev, St. Petersburg City AIDS Centre, Russian Federation; Andrew Phillips, University College London, UK, Kholoud Porter, University College London, UK; Claire Thorne, University College London, UK.

**EuroCoord Council of Partners**: Jean-Pierre Aboulker, Institut National de la Santé et de la Recherche Médicale, France; Jan Albert, Karolinska Institute, Sweden; Silvia Asandi, Romanian Angel Appeal Foundation, Romania; Geneviève Chêne, University of Bordeaux, France; Dominique Costagliola (chair), INSERM, France; Antonella d’Arminio Monforte, ICoNA Foundation, Italy; Stéphane De Wit, St. Pierre University Hospital, Belgium; Peter Reiss, Stichting HIV Monitoring, Netherlands; Julia Del Amo, Instituto de Salud Carlos III, Spain; José Gatell, Fundació Privada Clínic per a la Recerca Bíomèdica, Spain; Carlo Giaquinto, Fondazione PENTA, Italy; Osamah Hamouda, Robert Koch Institut, Germany; Igor Karpov, University of Minsk, Belarus; Bruno Ledergerber, University of Zurich, Switzerland; Jens Lundgren, Region Hovedstaden, Denmark; Ruslan Malyuta, Perinatal Prevention of AIDS Initiative, Ukraine; Claus Møller, Cadpeople A/S, Denmark; Kholoud Porter, University College London, United Kingdom; Maria Prins, Academic Medical Centre, Netherlands; Aza Rakhmanova, St. Petersburg City AIDS Centre, Russian Federation; Jürgen Rockstroh, University of Bonn, Germany; Magda Rosinska, National Institute of Public Health, National Institute of Hygiene, Poland; Manjinder Sandhu, Genome Research Limited; Claire Thorne, University College London, UK; Giota Touloumi, National and Kapodistrian University of Athens, Greece; Alain Volny Anne, European AIDS Treatment Group, France.

**EuroCoord External Advisory Board**: David Cooper, University of New South Wales, Australia; Nikos Dedes, Positive Voice, Greece; Kevin Fenton, Public Health England, USA; David Pizzuti, Gilead Sciences, USA; Marco Vitoria, World Health Organisation, Switzerland.

**EuroCoord Secretariat**: Silvia Faggion, Fondazione PENTA, Italy; Lorraine Fradette, University College London, UK; Richard Frost, University College London, UK; Andrea Cartier, University College London, UK; Dorthe Raben, Region Hovedstaden, Denmark; Christine Schwimmer, University of Bordeaux, France; Martin Scott, UCL European Research & Innovation Office, UK.
